# The Role of Ultrasound in the Evaluation of Elbow Medial Ulnar Collateral Ligament Injuries in Throwing Athletes

**DOI:** 10.1007/s12178-022-09793-0

**Published:** 2022-11-12

**Authors:** Brian J. Sutterer, Brennan J. Boettcher, Jeffrey M. Payne, Christopher L. Camp, Jacob L. Sellon

**Affiliations:** 1 Department of Physical Medicine and Rehabilitation, Mayo Clinic, 200 1st ST SW, Rochester, MN 55905 USA; 2 Departments of Orthopedic Surgery and Physical Medicine and Rehabilitation, Mayo Clinic, 200 1st ST SW, Rochester, MN 55905 USA; 3 Department of Orthopedic Surgery, Mayo Clinic, 200 1st ST SW, Rochester, MN 55905 USA

**Keywords:** Ultrasound, Ulnar collateral ligament, Throwing athletes, Medial ulnar collateral ligament

## Abstract

**Purpose of Review:**

Although ultrasound (US) imaging is commonly used to evaluate the elbow medial ulnar collateral ligament (mUCL) in throwing athletes, significant technical heterogeneity exists in the published literature and in practice. This has resulted in variable and often ambiguous US diagnostic criteria for mUCL injury. This review summarizes the literature on sonographic evaluation of the mUCL and outlines recommendations for consistent descriptive terminology, as well as future clinical and research applications.

**Recent Findings:**

Both acute and chronic throwing loads in overhead athletes cause the mUCL to become thicker and more lax on stress testing, and these changes tend to revert after a period of prolonged rest. Stress US (SUS) can aid in the diagnosis of mUCL tears and may help identify athletes at risk of mUCL injury. Variability exists in terminology, elbow flexion angle, amount of stress applied, and technique of stress testing. Recent studies have suggested an injured elbow stress delta (SD—change in ulnohumeral joint (UHJ) space with valgus stress) of 2.4 mm and a stress delta difference (SDD—side-side difference in SD) of 1 mm each denote abnormal UHJ laxity due to mUCL injury.

**Summary:**

US imaging is a powerful and widely accessible tool in the evaluation elbow mUCL injuries. Sonologists should consider how their US techniques compare with published methods and use caution when applying diagnostic criteria outside of those circumstances. Currently, an SD of 2.4 mm and an SDD of 1 mm provide the best diagnostic accuracy for mUCL tears requiring surgery. Finally, preliminary work suggests that shear wave elastography may be helpful in evaluating the biomechanical properties of the mUCL, but additional research is needed.

## Introduction

**Fig. 1:**
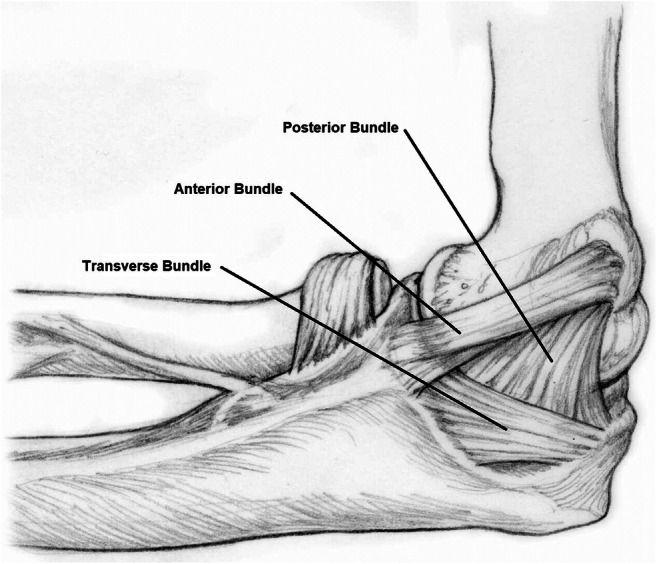
Anatomy of the mUCL

The medial ulnar collateral ligament (mUCL) of the elbow, specifically the anterior bundle, is the primary passive restraint to valgus stress on the elbow (Fig. 1) [1–3]. Repetitive stress from overhead throwing can lead to chronic overuse injuries of the mUCL and eventual partial or complete tearing [4]. These injuries most often occur in baseball pitchers but have also been reported in overhead athletes, such as football quarterbacks [5], javelin throwers [6], and softball players [7]. The incidence of mUCL injuries is increasing, highlighting the importance of gaining better understanding of mUCL structure and function and improving diagnostic assessment [8•, 9]. Diagnosis of mUCL injuries relies on an accurate history and physical exam. Patients will typically report acute on chronic medial elbow pain or a single episode of sudden onset pain and an associated “pop” after a throw. On physical exam, patients often have tenderness along the medial elbow at or near the mUCL origin with positive provocative testing, including the moving valgus stress test and milking maneuver. The diagnosis is aided by various imaging modalities, including static radiographs [10–13], stress radiographs [13–16], computed tomography (CT) [17], magnetic resonance imaging (MRI) [4, 17, 18, 19, 20, 21, 22••, 23, 24, 25, 26, 27], and ultrasound (US) [28–30]. US has been shown to be a low cost, efficient, and non-invasive method to evaluate the mUCL in throwing athletes. The unique capabilities of US in mUCL evaluation allow for direct visualization of pathology, dynamic assessment of ligament function, and measurement of tissue elasticity.

This review of currently available research aims to outline the various roles of US in evaluation of the mUCL in throwing athletes, particularly baseball players. We intend to identify and clarify existing terminology, review mUCL evaluation with stress ultrasound (SUS), identify morphologic and structural changes in response to load, consider the utility of US for injury prediction in overhead athletes, and discuss the potential role of US shear wave elastography (SWE) in mUCL assessment. We will also discuss limitations in the current literature and provide recommendations based on our collective clinical experience managing athletes with mUCL injuries.

## Evaluation of mUCL Function with Stress Ultrasound

**Fig. 2:**
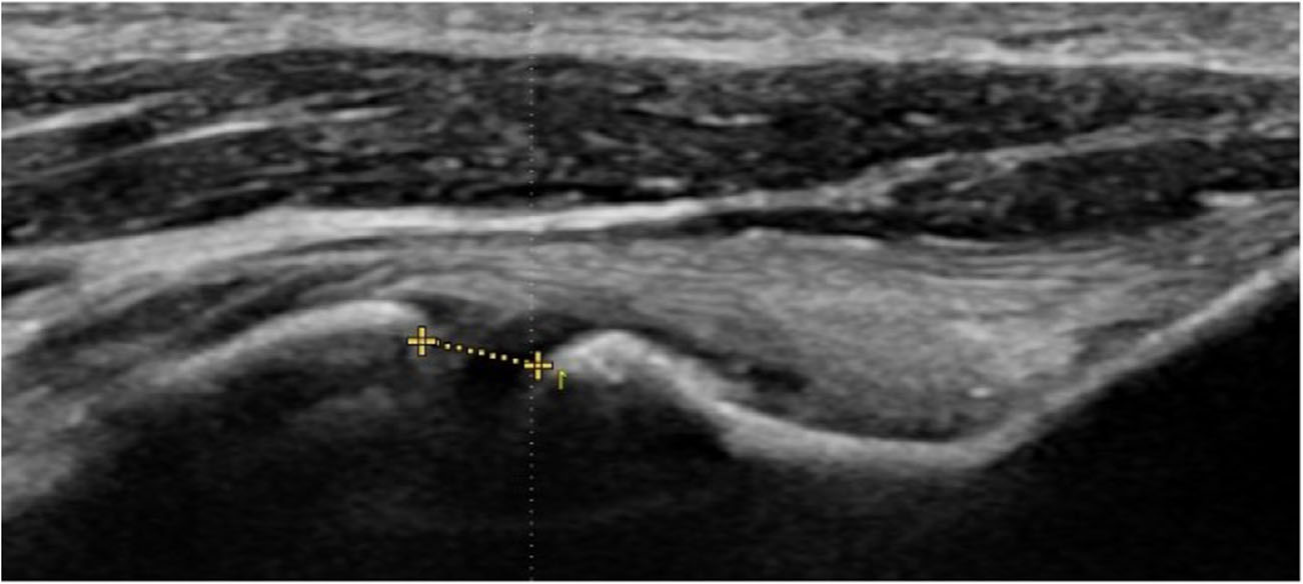
Ultrasound appearance of the mUCL with UHJ space noted with a dashed line. Right side is proximal

One of the primary benefits of US is the ability to perform dynamic evaluation of mUCL function under stress to gauge ulnohumeral joint (UHJ) laxity related to mUCL insufficiency or tearing. During SUS, the elbow is placed into flexion and the medial UHJ space (width) deep to the mUCL is measured at baseline and under valgus stress to replicate the functional load on the elbow during throwing (Fig. 2). When measuring the UHJ space, it is vitally important to mark the same locations on the humeral and ulnar sides of the joint for both the baseline and stress measurements. This can be done by capturing a cine loop during the stress maneuver and then scrolling through the frames to choose the best baseline and stress images for measurement. If US split screen is available, one can freeze the baseline image in one window and then use the other window to match the bony contours for the stress image. In our experience, the split screen technique facilitates more consistent marking of the joint margins.

**Fig. 3:**
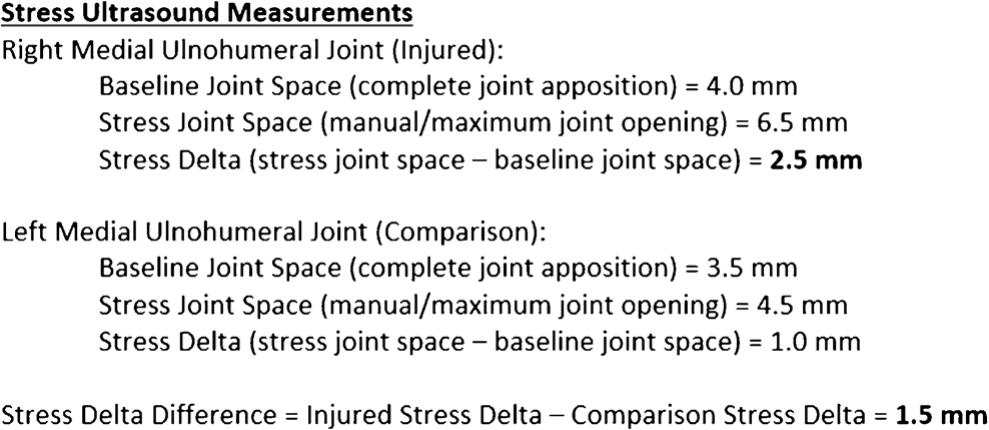
Sample stress ultrasound report

For the sake of this review and future work, we recommend the term *stress delta* (SD) to describe the change in joint space between stress and baseline conditions. We use the term *stress delta difference* (SDD) to describe the relative difference between the injured elbow stress delta (ISD) and the uninjured or comparison elbow stress delta (CSD), where SDD = ISD – CSD. Figure 3 shows an example of how these data can be summarized within an ultrasound report.

The amount of joint widening is related to the ligament integrity as demonstrated in cadaveric studies with sequential ligament sectioning [28, 31] and clinical studies of athletes with confirmed mUCL tears [22••, 32–34]. Multiple SUS techniques have been used in previous studies and have included a variety of force loads and flexion angles while applying stress. As such, several suggested criteria for what constitutes an abnormal exam exist (Table 1). Herein we intend to summarize the different techniques, forces, flexion angles, and criteria described, and provide our recommendations for incorporation of SUS into the evaluation of injured overhead athletes.

**Table 1:** Stress ultrasound criteria for abnormal ulnohumeral joint widening

*Study*	*Subjects*	*Method*	*Valgus load*	*flexion angle*	*Suggested criteria (tear description)*	*Sensitivity*	*Specificity*
Ciccotti et al [38] (2014)	Cadaver	Telos	150 N	30°	SD 1.4 mm (complete sectioning of anterior bundle)	-	-
Hendawi et al [51] (2019)	Cadaver	Telos	150 N	30°	SD 1.7 mm (complete sectioning of anterior bundle)	-	-
Ciccotti et al [31] (2020)	Cadaver	Telos	150 N	30°	SD 1.5 mm (complete sectioning of anterior bundle)	-	-
Roedl et al [22••] (2016)	144 baseball players	two-operator	manual max valgus stress	30°	SD 2.4 mm SDD 1 mm (tears requiring surgery)	SD: 87% SDD: 96%	SD: 86% SDD: 81%
Park et al [32] (2020)	137 throwing and 9 non-throwing athletes	two-operator	25 N	30°, 90°	SDD 0.5 mm at 30° SDD 1 mm at 90° (‘complete’ tears)	30° SDD: 88.1% 90° SDD: 81%	30° SDD: 61.5% 90° SDD: 66.4%
Gustas et al [52] (2016)	*Review*	None suggested			SDD 1.5 mm (‘full thickness’ tears)	SDD: 81%	SDD: 91%
Campbell et al [28] (2020)	*Review*	None suggested			SDD 1 mm (both partial and complete tears)	SDD: 96%	SDD: 81%
Ciccotti et al [29] (2020)	*Review*	None suggested			SD 1.5 mm (mUCL injury)	-	-
Hultman et al [53] (2021)	*Review*	-	150 N	30°	SD 2 mm SDD 1 mm (tears requiring surgery)	-	-

*SD* stress delta (injured elbow); *SDD* stress delta difference

### Amount and Application of Valgus Stress

**Fig. 4:**
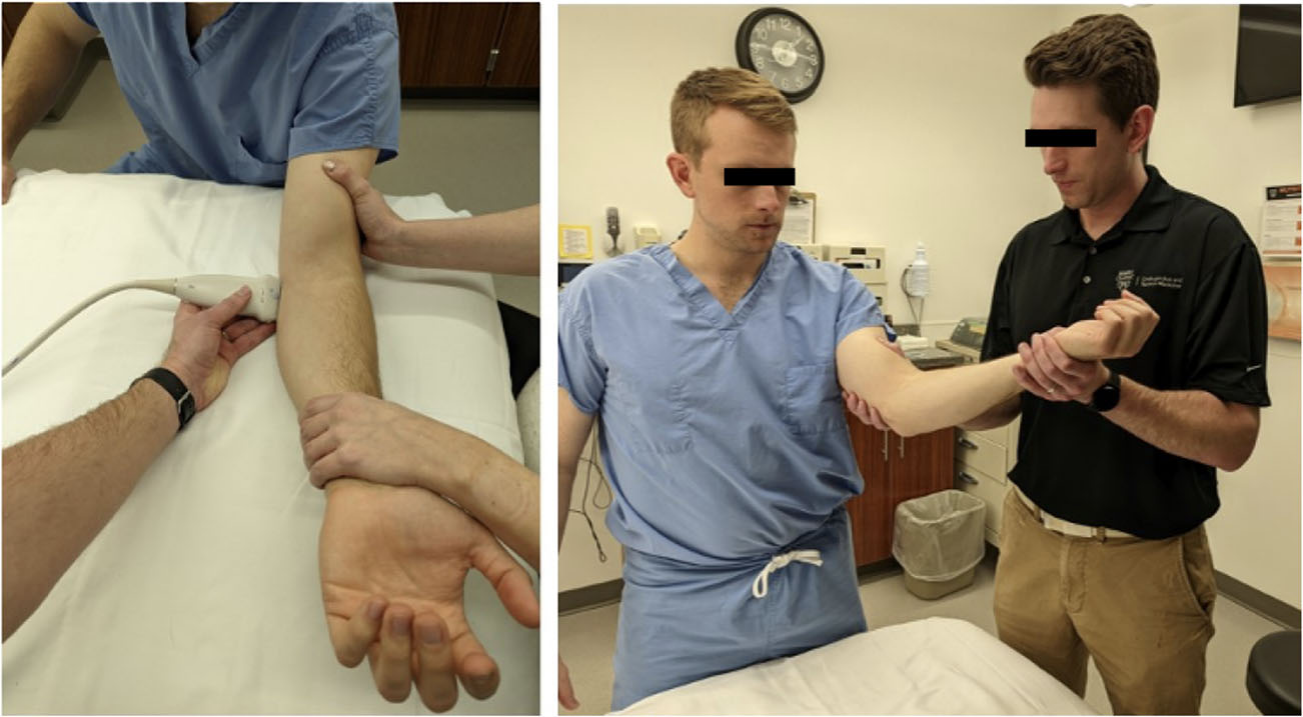
Two-operator SUS techniques in which an assistant applies a maximum valgus stress while the subject is either seated or standing, and the primary operator evaluates the mUCL with US

**Fig. 5:**
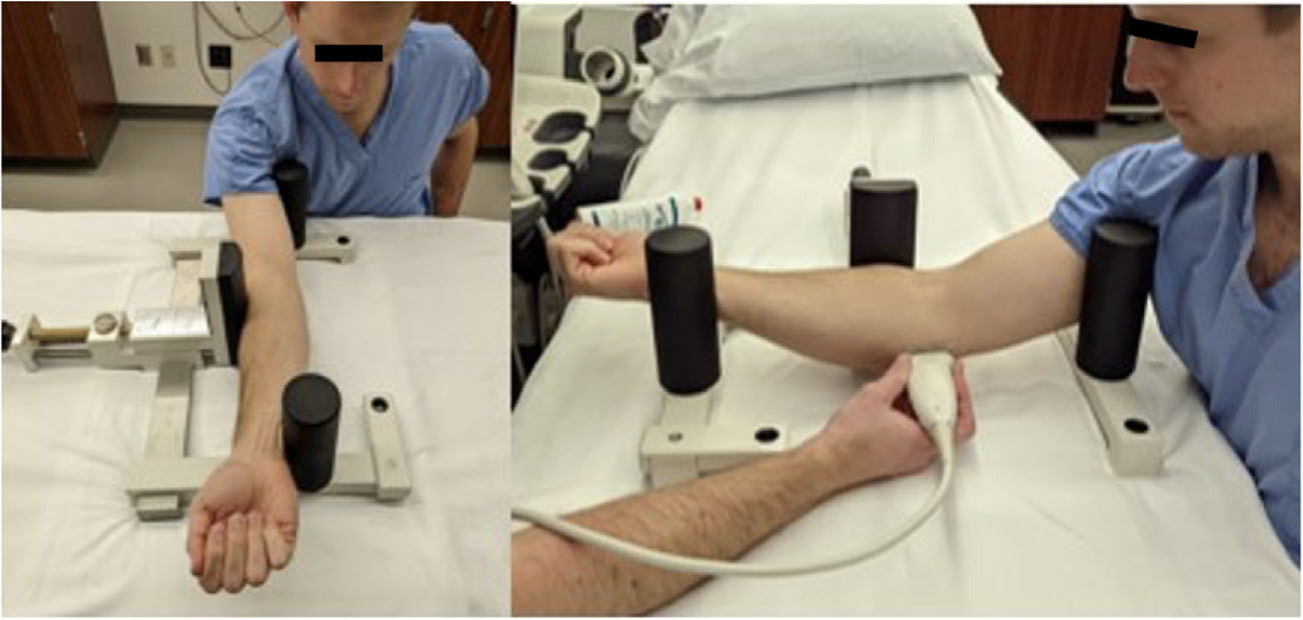
Telos stress testing device method of performing SUS. A manual crank is turned to increase the valgus force on the elbow while a digital readout shows the amount of force being applied

**Fig. 6:**
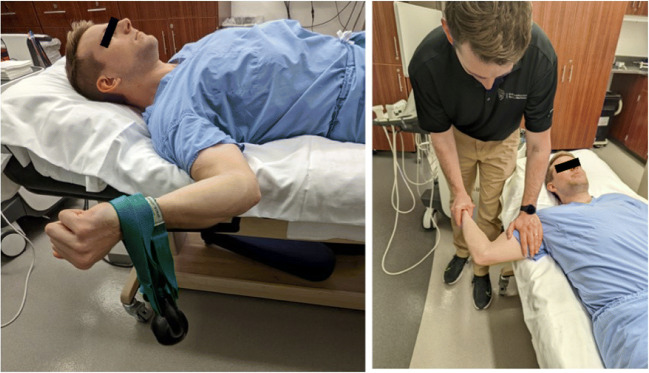
SUS techniques in which the subject lies supine, and a weight is either strapped to the wrist to provide a valgus stress, or a second operator applies a valgus stress from a standing position

In 2003, Ward et al. [35] characterized the sonographic appearance of the mUCL with 25 Newtons (N) of valgus force in healthy, non-throwing athletes. The earliest studies using SUS to evaluate for joint widening were two-operator techniques using a “maximum valgus stress” manually provided by an assistant [34, 36] or gravity stress alone [37] (Fig. 4). The first quantification of valgus load for SUS measurement of joint widening appears to be in 2014 in cadaveric and clinical studies by Ciccotti et al [38, 39•]. A 150 N force was applied to the elbow using a standardized stress device first used in stress radiograph studies (Telos SE 2000, METAX GmbH) (Fig. 5). The rationale for this amount of force was based on the first study of elbow stress radiography by Rijke et al in 1994 [16]. They measured UHJ widening at baseline and with 150 N of valgus load, showing that stress radiography enabled accurate diagnosis of mUCL tears. However, there was no justification given for selection of the amount of force used and no other forces were tested. Other studies have used a force of 25 N by either having subjects lie supine and hold a 2.5-kg weight or using a handheld dynamometer to measure a manually applied force (Fig. 6) [32, 40, 41•, 42]. This rationale is derived from a 1998 stress radiography study that used this force after a pilot study with a spring-loaded fish scale determined the manual force from a second operator providing maximum valgus stress was approximately 25 N [43].

**Fig. 7:**
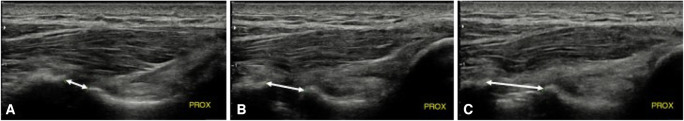
Medial UHJ space on SUS with **A** gravity eliminated (baseline), **B** gravity stress alone, and **C** manual valgus stress to point of maximum UHJ widening

To our knowledge, no studies have evaluated how UHJ space changes during SUS with increasing valgus force in patients with mUCL tears. In uninjured college students, Ikuta et al. showed a joint space of 3.95 mm under 30 N compared to 4.25 mm under 60 N, measured at 30 degrees of elbow flexion [44]. In interpreting such studies, it is important to consider the potential for false negative results due to compensatory wrist flexor-pronator muscle activation that may help stabilize the elbow in the setting of mUCL injury. Prior work with stress radiographs and 150 N of force found that some individuals with mUCL injuries actually display *less* joint widening under stress compared to the uninjured side, with reflexive guarding with muscle contraction a suggested explanation for this contradictory finding [13]. Yoshioka et al [45••] used surface EMG to show that dynamic muscle activation of the flexor-pronator mass started above 60 N of force and suggested this as an optimal valgus stress to evaluate the mUCL function without risking a possible false negative effect related to compensatory muscle contraction. Sonologists who perform SUS in a medial elbow up position, as may be the case in a supine or side-lying position, should also be aware that the force of gravity alone can artificially increase the baseline UHJ space (Fig. 7). Based on our experience, it is essential that the elbow is in a neutral or gravity-eliminated position when the baseline UHJ space is measured. The force of gravity can be counteracted with light varus stress to the point of maximal UHJ apposition.

Further research is needed to assess how force variation affects SUS diagnostic accuracy in the evaluation of mUCL tears. It is also important to consider patient tolerance to the procedure and the possible confounding effect of muscle contraction if the force is unnecessarily high. It is not clear whether published diagnostic criteria remain valid across different testing methods and valgus loads, and sonologists should accordingly use caution in applying these standards. Based on current knowledge, we recommend operators use a force of 60 N or less when performing mUCL testing. In our experience, if testing is performed using manual valgus stress with the patient in a side-lying position, a force of 15–25 N is sufficient to obtain maximal UHJ opening and is generally well tolerated. If a handheld dynamometer or weight is not available, a manual valgus force can be applied until no further joint widening is seen on US. When using a Telos device, it is important to know that diagnostic criteria have been established only with 150 N of force, while also considering the aforementioned study indicating that force greater than 60 N may trigger false negative results via compensatory muscle contraction [45••].

### Flexion Angle

Most SUS studies have been performed using either 30° or 90° of elbow flexion. These angles have been rationalized with various justifications. Biomechanics studies of cadaveric specimens with transected mUCLs have evaluated the amount of valgus instability with a constant force at different flexion angles, but this has yielded some inconsistency. Callaway et al [1] found the greatest instability at 90°, while Floris et al [46] and Søjbjerg et al [47] found laxity to be the greatest at 70°. Recently, Rogers et al. found maximum UHJ instability at 49° by using a robot to impart valgus force through a full range of motion in mUCL deficient cadaveric elbows [48•].

The earliest case report using SUS for evaluation of mUCL injury used 25° of elbow flexion [34]. Shortly thereafter, Nazarian et al. [36] and Sasaki et al. [37] used elbow flexion angles of 30° and 90°, respectively, in their SUS studies. The Nazarian study chose 30° based on the point at which the olecranon is unlocked from its fossa and the mUCL becomes the primary valgus stabilizer, while Sasaki et al. did not provide rationale for selecting 90°. Subsequent work by Ciccotti et al in 2014, studying both cadaver specimens and a large population of professional pitchers, used 30° of flexion [38, 39•]. While they acknowledged 90° better replicates the maximum stress position in an overhead throw, they chose 30° based on prior biomechanics work by Morrey et al [2] and the positioning limitations of the Telos stress device used to apply the valgus load. More recently, studies have evaluated the mUCL with the elbow at 90° of flexion to reproduce the elbow position during maximal stress in throwing [32, 41•, 42, 49, 50].

There is little clinical data comparing elbow positions during SUS. Park et al. [32] studied subjects with mUCL tears at both 30° and 90° of elbow flexion. Receiver operator characteristic (ROC) curves showed sensitivity and specificity for complete mUCL tears at 30° was 88.1% and 61.5% using an SDD threshold of 0.5 mm, while 90° resulted in 81% and 66.4% with an SDD of 1.0 mm. ROC areas were not significantly different, and the authors suggested using 90° based on 1 mm of difference being more reliable to detect. To our knowledge, this is the only study to evaluate live subjects with mUCL injuries at multiple angles, and no other flexion angles have been studied in injured athletes. Shanley et al. [41•] used a “moving valgus stress” to determine maximum joint widening but did not actually specify the elbow flexion angle where that occurred, beyond taking the baseline measurement at 90°.

Despite a growing body of literature, there is no consensus on the optimal elbow position during SUS. While 90° may approximate the typical throwing position better than 30°, it is not clear this translates to the best position for SUS testing of mUCL integrity. Most of the existing literature has studied SUS at 30°, and greater sensitivity and specificity have been demonstrated at this angle [22••]. However, it is important to note that SUS at elbow angles other than 30° and 90° remains largely uninvestigated. We are compelled by the aforementioned biomechanical study by Rogers et al. showing maximum instability in the mUCL deficient elbow *across the entire range of motion* to occur at about 50°, and we have found an angle of around 45 degrees works well when using our preferred single-operator, side-lying SUS technique. Nevertheless, based on the existing literature, we acknowledge 30° is currently the best validated elbow position for SUS. If other SUS elbow flexion angles are used, it is important to recognize that currently established abnormal criteria may not be valid.

### Criteria for Abnormal Findings

Cadaveric and clinical studies have suggested criteria for what amount of SD constitutes an “abnormal” finding (Table 1). There is significant variability in these criteria, likely related to heterogeneity in the testing methods as previously outlined. With regard to cadaveric investigations, Ciccotti et al. [38] performed mUCL sectioning of 12 cadaver elbows and recommended an SD of 1.4 mm or more as indicative of anterior bundle complete tear. A subsequent cadaveric study by this same author suggested > 1.5 mm as a recommended SD cutoff for clinical applications [31]. Finally, Hendawi et al. [51] found an SD of 1.7 mm after US-guided anterior bundle transection.

From a clinical perspective, Roedl et al. [22••] studied SUS in baseball players with mUCL tears requiring surgery and found that an injured elbow SD of >2.4 mm had a sensitivity of 87% and specificity of 86%, while an SDD of >1 mm resulted in sensitivity of 96% and specificity of 81%. In contrast, the aforementioned study by Park et al. in throwing athletes with complete mUCL tears, using the same 30° elbow angle but perhaps lower valgus force, found an SDD of >0.5 mm with sensitivity of 88.1% and specificity of 61.5%. Multiple review articles have proposed criteria for SD ranging from >1.5–2 mm and SDD ranging from >1 to 1.5 mm [28, 29, 52, 53].

Based on current evidence, we recommend using the criteria established by Roedl et al. [22••] (SD >2.4 mm or SDD >1 mm) as these correlated with surgical mUCL injuries. Regarding the commonly cited SD threshold of 1.5 mm [29], we think this is too similar to the SD commonly seen in uninjured throwing athletes. For example, Nazarian et al. and Shanley et al. found an SD of 1.4 mm and 1.3 mm, respectively, in professional pitchers without mUCL injuries [36, 41•]. It is also important to point out that valgus stress applied in these two studies was significantly less than the 150 N applied during the techniques used in recommending an SD threshold of 1.5 mm [29]. In another study of healthy pitchers after 100 sequential pitches, 1.4 mm of SD was observed with just 30 N of stress [54•]. Khalil et al. [55] found 1.77 mm SD in healthy college pitchers after a full season. Therefore, the SD criteria of 1.5 mm may be too close to an expected normal range in uninjured athletes.

### Additional Role in Diagnosis and Management of mUCL Tears

Despite advancements in ultrasound techniques, MRI remains the gold standard in diagnosing mUCL tears. However, there are several benefits to adding US to the diagnostic evaluation. The addition of US to MRI arthrogram alone for diagnosing mUCL tears increased sensitivity from 81% to 96% and specificity from 91% to 99%, respectively [22••]. US can also help differentiate normal adaptive changes from a functionally incompetent ligament. Tanaka et al. [56] performed screening MRIs on 64 asymptomatic pitchers and found 34 showed evidence of an mUCL injury, yet there was no correlation with ligament dysfunction based on SUS joint widening. The authors theorized that abnormal MRI findings were suggestive of chronic adaption of the mUCL, highlighting the diagnostic value of adding dynamic US evaluation of ligament function.

**Fig. 8:**
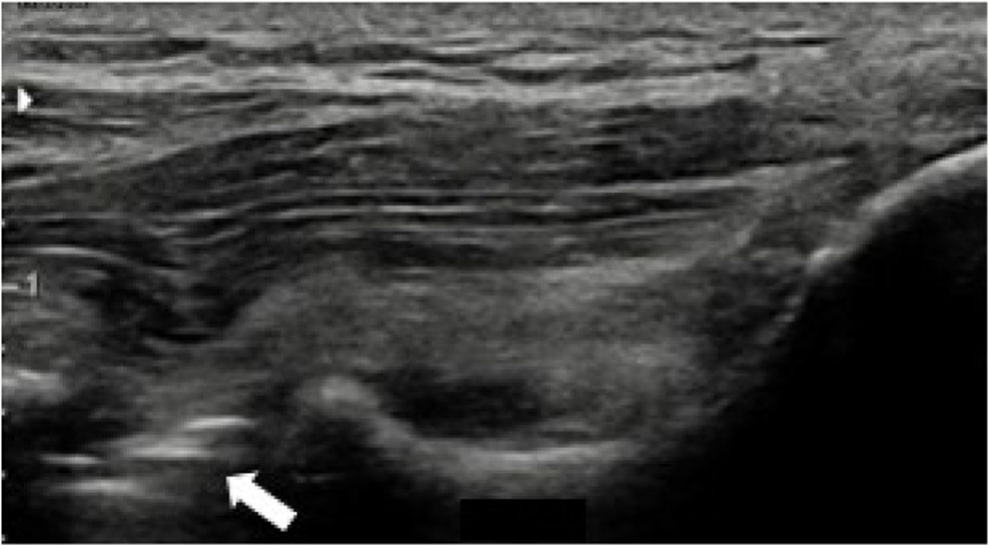
Ring-down artifact (arrow) in a patient with a full-thickness mUCL tear and significant UHJ widening under stress. Right side is proximal

US has also been shown to help predict the rehabilitation outcome of mUCL injuries. Kim et al. retrospectively evaluated a cohort of 41 baseball players with mUCL injuries who initially completed 6 weeks of non-operative management before 18 ultimately required surgery [33]. They used US to evaluate joint laxity, tenderness, and the presence of an echogenic “ring-down” artifact in the UHJ deep to the mUCL, presumed to result from gas bubbles generated in the joint cavity after ligament injury due to a transient vacuum phenomenon (Fig. 8) [57]. The ring-down artifact was significantly more prevalent in the surgery group, and the authors concluded that addition of US to conventional MRI allowed for improved differentiation between the two groups.

US imaging also plays a role in the evaluation of partial and chronic mUCL tears. Whereas 3T MRI and MR arthrography have been shown to be highly sensitive and specific for full-thickness (presumed complete) tears by Magee et al [21], conventional MRI has shown variable results in smaller studies [22••, 58]. In chronic and partial injuries, the degree of abnormal signal and MRI findings depends on the chronicity and severity of injury. Ligament thickening and morphologic changes that may appear abnormal on MRI may be a normal adaptation from repetitive loading. The addition of US, including SUS to assess functional laxity, can help distinguish normal adaptions from pathologic injuries. Roedl et al. [22••] found US had a sensitivity and specificity of 77% and 94% for detecting partial mUCL tears, compared to 74% and 92%, respectively, using MR arthrography alone.

## Assessment of mUCL Morphology and Change with Load

As noted above, US can be used to evaluate the morphology of the mUCL in throwing athletes, both in the monitoring of normal adaptive response to load, but also in the assessment of mUCL injury or other causes of elbow pain. Compared to MRI, US also has the distinct advantage of easy access to an internal comparison, utilizing the contralateral elbow to assess an athlete’s “normal.” In baseball players, multiple studies have demonstrated that the mUCL thickens and becomes more lax with repetitive load [39•, 42, 59, 60]. Other work by Khalil et al. [55] found that while mUCL thickness significantly increased after a season of collegiate pitching, joint laxity did not. Studies that reevaluated the mUCL after a period of offseason rest have found that it becomes thinner and less lax [42, 61]. Even a single throwing outing has also been shown to influence ligament properties. High school baseball pitchers demonstrated significant amounts of joint widening from baseline after just 60 successive pitches [54•]. In another study of 100 successive pitches in a similar population of throwing athletes, mUCL stiffness significantly decreased on SWE [50]. A common guideline for youth pitching limits is 100 pitches per outing; however, these data suggest that stress-related changes from repetitive loading of the mUCL can actually occur at lower pitch counts, and US may be helpful in early recognition of this excessive mUCL stress.

The thickness of the mUCL has been shown to be greater in older athletes and those with more years of experience [39•, 60, 62]. The majority of studies comparing mUCL thickness between dominant and non-dominant elbows in throwing athletes have shown increased mUCL thickness in the dominant limb [36, 39•, 59, 61–65]. Increased hyperechoic foci and calcifications have also been found in the throwing elbow [36, 39•, 60]. A single study of high school pitchers showed similar thickness and calcification presence in the two sides, but with more hyperechoic foci in the dominant elbow [66]. In 2001, Popovic et al. [63] used US to determine the mUCL was significantly thicker in the throwing elbow of uninjured handball athletes. Finally, from a clinical perspective, another study found athletes with a history of elbow pain were found to have significantly greater mUCL thickness than those without [64].

**Fig. 9:**
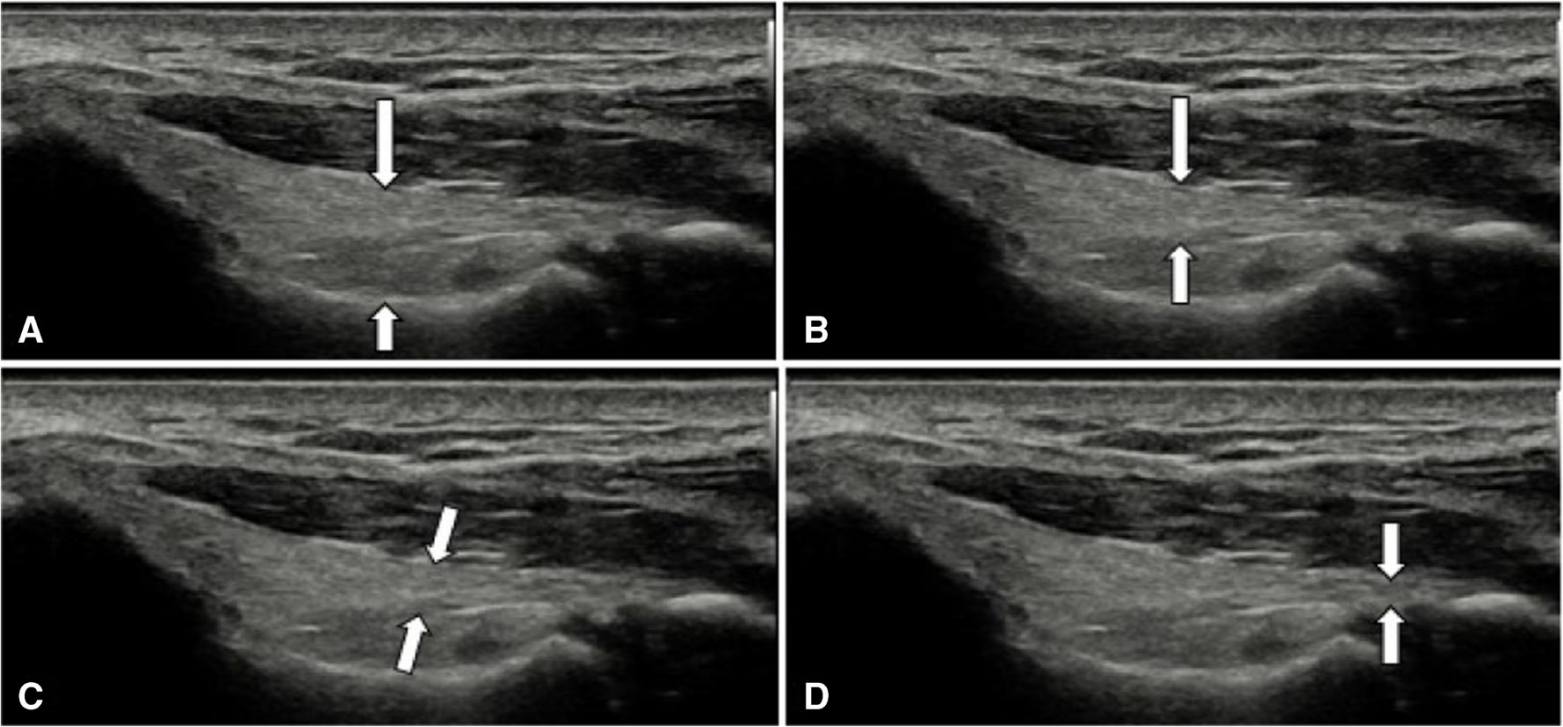
mUCL thickness measurement methods. **A** Nazarian, **B** Jacobson-Ward, **C** Modified Jacobson-Ward, **D** distal measurement

Four primary methods are described to measure the thickness of the mUCL (Fig. 9). Ward et al. [35] and Jacobsen et al. [67] described a technique measuring the midportion of the ligament without including the underlying fat pad, whereas Nazarian et al. [36] measured the thickness from the superficial aspect of the ligament at its midportion down to the underlying bone, including the fat pad. Shukla et al. [68] found the inter-rater reliability for the Nazarian technique was higher than the Jacobson-Ward technique (0.82 vs 0.51) and proposed a “modified Jacobson-Ward” technique, in which mUCL thickness is measured perpendicular to the ligament fibers and had an ICC of 0.84. Shanley et al. [41•] and Tajika et al. [64] measured thickness more distally over the trochlea, further adding to the heterogenity in existing data. Further research is needed comparing these methods in serial evaluations to ensure the morphologic changes are being appropriately measured. All four methods have been used separately in studies showing increased mUCL thickness with repetitive load, providing a level of assurance that any of them may be sufficient to capture changes.

## Injury Risk Evaluation

mUCL injuries can have a devastating impact on the career of a throwing athlete. Cain et al. [20] analyzed return to sport in 743 athletes from various sports and at all levels of competition, who underwent surgery after mUCL tears, finding that 17% were unable to return to their previous level of competition. A 2020 systematic review of professional baseball pitchers found return to the same level of play in 67–87% of athletes in around 15 months [69]. This impact highlights the need for consideration of injury risk factors and monitoring. Shanely et al. [41•] found that professional pitchers with a stress joint space of ≥5.6 mm when the elbow was stressed at 90° were at a 6 times greater risk of sustaining a mUCL tear requiring surgery within the next season. This is in contrast to a prior 10-year study of professional pitchers by Ciccotti et al. [39•] that did not find a significant relationship between SUS findings and future mUCL injury risk, despite a trend towards increased mUCL thickness and joint widening under stress in the athletes with tears. One possible reason for the different findings is the difference in SUS techniques. Shanley et al. used 25 N of force with the elbow flexed at 90°, whereas Ciccotti et al. applied 150 N to the elbow at 30°. As previously discussed, reflexive activation of the flexor-pronator mass has been shown beyond 60 N of force at 30° [45••]. Therefore, it is possible subjects in the Ciccotti et al. study subjected to higher valgus stress may have had less joint widening due to compensatory muscle activation.

We were unable to find any published studies on the use of US in-season to assess injury risk. While Hattori et al. [54•] demonstrated that significant UHJ laxity on SUS occurs after just 60 pitches in high school athletes, it is not clear how long these effects last. Similarly, Ikuta at al. found evidence of a creep phenomenon when the mUCL was placed under a continuous valgus stress. UHJ space was significantly increased after 60 s of a continuous 30 N force and after 120 s of 60 N of force with a dose-response effect showing greater joint space with the higher force [44]. Future research is needed on the cumulative in-season changes in mUCL laxity and thickness in throwing athletes. This could provide valuable understanding of how injury risk might change as the mUCL responds to the fluctuation in load. The prior discussion suggests there may be utility in using US to monitor mUCL characteristics, and if critical measurements are reached, load could be reduced to lower injury risk.

## Ultrasound Elastography

**Fig. 10:**
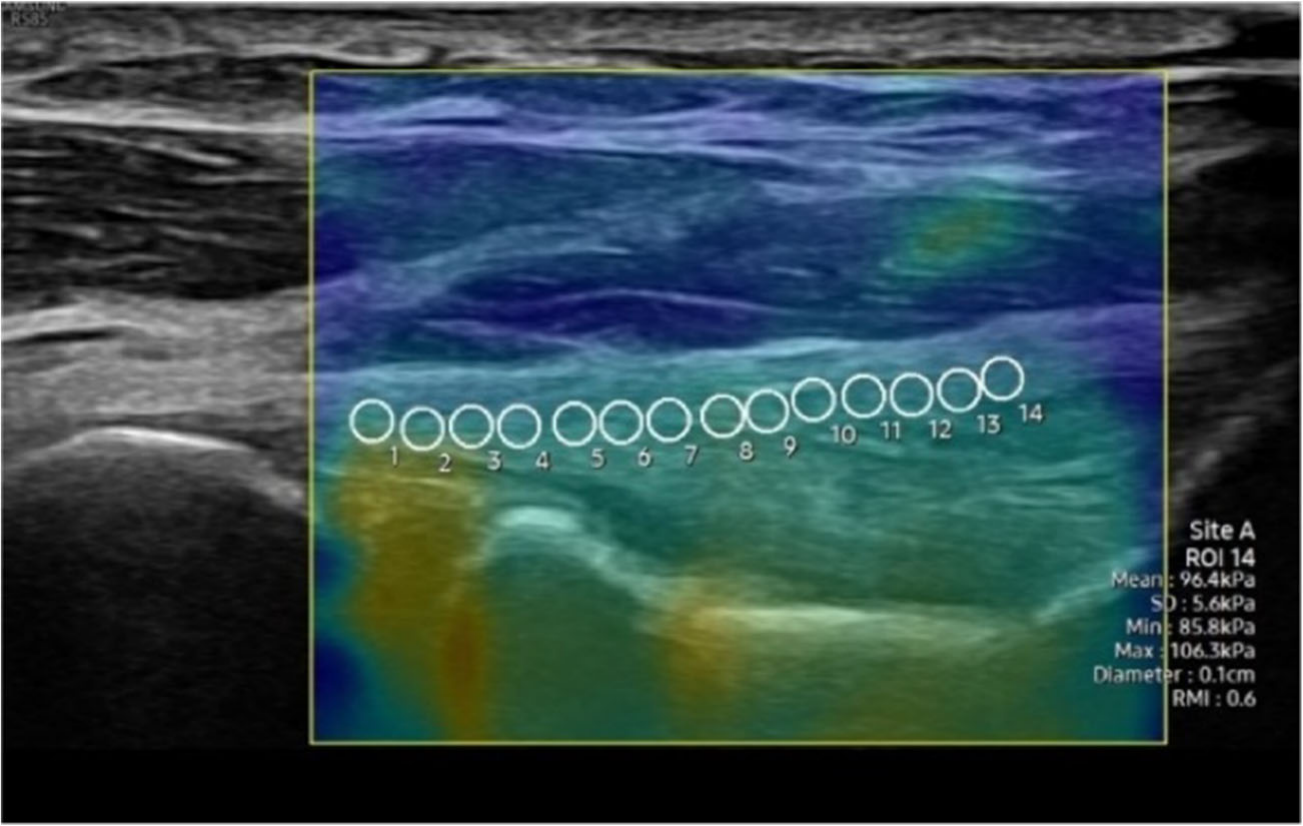
Example shear wave elastography of the mUCL

US SWE is a noninvasive imaging technique used to evaluate the mechanical properties of tissues and allows for a quantitative measurement of tissue stiffness. In SWE, an acoustic radiation force, sometimes referred to as an “acoustic wind,” produced by a linear array US transducer, generates shear waves that propagate perpendicular to the primary US waves and at a lower velocity [70]. As the shear waves propagate through tissue, shear wave speed (SWS) is measured based on the transient tissue displacements and a qualitative color map is produced. SWS can then be used to calculate shear modulus, or tissue elasticity, providing information about the tissue’s biomechanical properties [71]. A color map is generated over the area of interest, and the tissue properties can be measured from selected areas (Fig. 10). In musculoskeletal applications, SWE has primarily been used to evaluate muscles and tendons [70–73]. Previous studies on the Achilles, patellar tendon, and wrist extensor tendons have shown that pathologic tendons have lower stiffness and that SWE measurements correlate better with patient symptoms than conventional ultrasound findings [74].

Limited research has been performed using elastography to evaluate ligaments, including the mUCL. Lin et al. [75] performed SWE in 16 healthy, nonthrowing individuals and found an average shear modulus of 110.2 kPa. Intra-day reliability was good (ICC = 0.715) and day-to-day reliability was excellent (ICC = 0.984) [75]. These results were compared to a single baseball pitcher with a mUCL injury, using a shear wave modulus of 186.45 kPa on the injured side compared to 879.59 kPa* on the uninjured side. *Of note, our experience performing SWE in around 100 professional pitchers showed no values above 300 kPa, raising a question about the reproducibility of this result. Gupta et al. [76] reported a mean SWS in the dominant mUCL of males and females to be 5.35 m/s and 5.03 m/s, respectively, with poor reliability (ICC = 0.05). Two studies by Hattori et al. [49, 50] utilized a different type of elastography (strain elastography) to evaluate the mUCL in high school pitchers. In strain elastography, a transducer-induced perturbation exerts a small amount of pressure on the underlying tissue and the corresponding strain of the region of interest is compared to the surrounding tissue with a higher strain ratio indicating less elasticity. One study by Hattori et al. [49] found excellent reliability of this technique for evaluating the mUCL with an ICC of 0.91 and 0.83 for each examiner. In the other study, the mUCL strain ratio decreased after 100 pitches, indicating increased elasticity with repetitive short-term load [50].

Additional research is needed to better understand the clinical utility of US elastography for evaluation of the elbow mUCL. There are many limitations and challenges with this technology, including the heterogeneous nature of musculoskeletal tissues, the effects of transducer pressure and patient positioning, the depth of the tissue being evaluated, and variations between individual US machines [70, 73]. SWE is being investigated in various soft tissue pathology, in particular tendinopathy and acute tendon injuries, but there remains significant heterogeneity in the findings and reliability [70–73]. In our experience, SWE is more reliable than strain elastography, and we think SWE has greater potential for mUCL evaluation. With respect to the mUCL, there is limited research on SWE values in healthy subjects, and only a single case report of SWE in a throwing athlete with a mUCL injury. Future research on SWE of the mUCL in healthy and injured throwing athletes will help better define the role of this emerging technology.

## Summary and Future Recommendations

This review summarizes the current and emerging utility of US imaging, including SUS and SWE, in the evaluation of the mUCL in throwing athletes. However, it also highlights the significant variability in the methods of performing these evaluations. This is likely the main reason for inconsistencies in SUS findings and injury risk monitoring recommendations. Future studies are needed to directly compare these different methodologies and elucidate the optimal approach. In the future, US monitoring of mUCL injuries may play a role in assessing readiness for return to sports. However, there remains a large gap in the literature regarding US assessment of the mUCL outside of the acute phase of injury. While pitchers with a history of mUCL reconstruction are known to have thicker mUCLs and less UHJ widening on SUS compared to those with a native mUCL [77], there is no existing literature on the use of US to monitor progression and readiness during the post-operative rehabilitation period.

In summary, US imaging can be used to monitor the morphologic and functional changes of the mUCL in response to throwing load. The mUCL tends to be thicker and more lax in the dominant/throwing limb, with older age, with increased throwing load, and in athletes with elbow pain. SUS can aid the diagnosis and prognosis of acute and chronic mUCL injuries by determining the SD and SDD, but more comparative studies are needed to optimize SUS technique and establish better diagnostic criteria. There is conflicting evidence to date regarding the ability of US to predict mUCL injury risk in pitchers, and more research is needed. Finally, SWE may play an increasing role in the evaluation and prevention of mUCL injuries in throwing athletes.

## References

[1] Callaway GH, Field LD, Deng XH, Torzilli PA, O'Brien SJ, Altchek DW (1997). Biomechanical evaluation of the medial collateral ligament of the elbow. J Bone Joint Surg Am..

[2] Morrey BF, Tanaka S, An KN (1991). Valgus stability of the elbow. A definition of primary and secondary constraints. Clin Orthop Relat Res.

[3] Kaufmann RA, Wilps T, Musahl V, Debski RE (2020). Elbow biomechanics: soft tissue stabilizers. J Hand Surg Am..

[4] Joyner PW, Bruce J, Hess R, Mates A, Mills FB, Andrews JR (2016). Magnetic resonance imaging-based classification for ulnar collateral ligament injuries of the elbow. J Shoulder Elbow Surg..

[5] Dodson CC, Slenker N, Cohen SB, Ciccotti MG, DeLuca P (2010). Ulnar collateral ligament injuries of the elbow in professional football quarterbacks. J Shoulder Elbow Surg..

[6] Dines JS, Jones KJ, Kahlenberg C, Rosenbaum A, Osbahr DC, Altchek DW (2012). Elbow ulnar collateral ligament reconstruction in javelin throwers at a minimum 2-year follow-up. Am J Sports Med..

[7] Dugas J, Chronister J, Cain EL, Andrews JR (2014). Ulnar collateral ligament in the overhead athlete: a current review. Sports Med Arthrosc Rev..

[8.•] Leland DP, Conte S, Flynn N, Conte N, Crenshaw K, Wilk KE, et al. Prevalence of Medial Ulnar Collateral Ligament Surgery in 6135 Current Professional Baseball Players: A 2018 Update. Orthop J Sports Med. 2019;7(9):2325967119871442. 10.1177/2325967119871442. **The most recent data on the prevalance of mUCL injuries in professional baseball players based on survey data from the 2018 season. This found an overall prevalance of mUCL reconstruction of 13%, and 20% in just pitchers. The overall prevalance was increased from 10% in 2012.** 10.1177/2325967119871442 PMC6764054 31598529

[9] Erickson BJ, Nwachukwu BU, Rosas S, Schairer WW, McCormick FM, Bach BR (2015). Trends in medial ulnar collateral ligament reconstruction in the United States: a retrospective review of a large private-payer database from 2007 to 2011. Am J Sports Med..

[10] Salvo JP, Rizio L, Zvijac JE, Uribe JW, Hechtman KS (2002). Avulsion fracture of the ulnar sublime tubercle in overhead throwing athletes. Am J Sports Med..

[11] Petty DH, Andrews JR, Fleisig GS, Cain EL (2004). Ulnar collateral ligament reconstruction in high school baseball players: clinical results and injury risk factors. Am J Sports Med..

[12] Mulligan SA, Schwartz ML, Broussard MF, Andrews JR (2000). Heterotopic calcification and tears of the ulnar collateral ligament: radiographic and MR imaging findings. AJR Am J Roentgenol..

[13] Bruce JR, Hess R, Joyner P, Andrews JR (2014). How much valgus instability can be expected with ulnar collateral ligament (UCL) injuries? A review of 273 baseball players with UCL injuries. J Shoulder Elbow Surg..

[14] Azar FM, Andrews JR, Wilk KE, Groh D (2000). Operative treatment of ulnar collateral ligament injuries of the elbow in athletes. Am J Sports Med..

[15] Molenaars RJ, Medina GIS, Eygendaal D, Oh LS (2020). Injured vs. uninjured elbow opening on clinical stress radiographs and its relationship to ulnar collateral ligament injury severity in throwers. J Shoulder Elbow Surg..

[16] Rijke AM, Goitz HT, McCue FC, Andrews JR, Berr SS (1994). Stress radiography of the medial elbow ligaments. Radiology..

[17] Timmerman LA, Schwartz ML, Andrews JR (1994). Preoperative evaluation of the ulnar collateral ligament by magnetic resonance imaging and computed tomography arthrography. Evaluation in 25 baseball players with surgical confirmation. Am J Sports Med..

[18] Ramkumar PN, Frangiamore SJ, Navarro SM, Lynch TS, Forney MC, Kaar SG, Akhavan S, Moutzouros V, Westermann RW, Farrow LD, Schickendantz MS (2018). Interobserver and intraobserver reliability of an MRI-Based classification system for injuries to the ulnar collateral ligament. Am J Sports Med..

[19] Ciccotti MC, Stull JD, Buckley PS, Cohen SB (2017). Correlation of MRI to arthroscopy in the elbow: thrower's elbow and ulnar collateral ligament injury. Sports Med Arthrosc Rev..

[20] Cain EL, Andrews JR, Dugas JR, Wilk KE, McMichael CS, Walter JC (2010). Outcome of ulnar collateral ligament reconstruction of the elbow in 1281 athletes: results in 743 athletes with minimum 2-year follow-up. Am J Sports Med..

[21] Magee T (2015). Accuracy of 3-T MR arthrography versus conventional 3-T MRI of elbow tendons and ligaments compared with surgery. AJR Am J Roentgenol..

[22.••] Roedl JB, Gonzalez FM, Zoga AC, Morrison WB, Nevalainen MT, Ciccotti MG, et al. Potential Utility of a Combined Approach with US and MR Arthrography to Image Medial Elbow Pain in Baseball Players. Radiology. 2016;279(3):827-37. 10.1148/radiol.2015151256. **The most accurate cutoff on stress ultrasound for mUCL tears requiring surgery was an injured stress delta (ISD) of 2.4 mm and a stress delta difference (SDD) of 1 mm. The sensitivity and specifity for this ISD was 87% and 86%, respectively. The sensitivity and specificity for this SDD was 96% and 81%, respectively.** 10.1148/radiol.2015151256 27183408

[23] Fowler KA, Chung CB (2006). Normal MR imaging anatomy of the elbow. Radiol Clin North Am..

[24] Hill NB, Bucchieri JS, Shon F, Miller TT, Rosenwasser MP (2000). Magnetic resonance imaging of injury to the medial collateral ligament of the elbow: a cadaver model. J Shoulder Elbow Surg..

[25] Timmerman LA, Andrews JR (1994). Undersurface tear of the ulnar collateral ligament in baseball players. A newly recognized lesion. Am J Sports Med..

[26] Schwartz ML, al-Zahrani S, Morwessel RM, Andrews JR. Ulnar collateral ligament injury in the throwing athlete: evaluation with saline-enhanced MR arthrography. Radiology. 1995;197(1):297-299. 10.1148/radiology.197.1.7568841. 10.1148/radiology.197.1.7568841 7568841

[27] Nakanishi K, Masatomi T, Ochi T, Ishida T, Hori S, Ikezoe J, Nakamura H (1996). MR arthrography of elbow: evaluation of the ulnar collateral ligament of elbow. Skeletal Radiol..

[28] Campbell RE, McGhee AN, Freedman KB, Tjoumakaris FP (2020). Diagnostic imaging of ulnar collateral ligament injury: a systematic review. Am J Sports Med..

[29] Ciccotti MC, Ciccotti MG (2020). Ulnar collateral ligament evaluation and diagnostics. Clin Sports Med..

[30] Dixit A, Dandu N, Hadley CJ, Nazarian LN, Cohen SB, Ciccotti M. ultrasonographic technique, appearance, and diagnostic accuracy for common elbow sports injuries. JBJS Rev. 2020;8(11):e19.00219. 10.2106/jbjs.Rvw.19.00219. 10.2106/JBJS.RVW.19.00219 33186208

[31] Ciccotti MC, Hammoud S, Dodson CC, Cohen SB, Nazarian LN, Ciccotti MG (2020). Medial elbow instability resulting from partial tears of the ulnar collateral ligament: stress ultrasound in a cadaveric model. Am J Sports Med..

[32] Park JY, Kim H, Lee JH, Heo T, Park H, Chung SW, Oh KS (2020). Valgus stress ultrasound for medial ulnar collateral ligament injuries in athletes: is ultrasound alone enough for diagnosis?. J Shoulder Elbow Surg..

[33] Kim NR, Moon SG, Park JY, Choi JW, Oh KS (2017). Stress ultrasound in baseball players with ulnar collateral ligament injuries: additional value for predicting rehabilitation outcome. J Shoulder Elbow Surg..

[34] De Smet AA, Winter TC, Best TM, Bernhardt DT (2002). Dynamic sonography with valgus stress to assess elbow ulnar collateral ligament injury in baseball pitchers. Skeletal Radiol..

[35] Ward SI, Teefey SA, Paletta GA, Middleton WD, Hildebolt CF, Rubin DA, Yamaguchi K (2003). Sonography of the medial collateral ligament of the elbow: a study of cadavers and healthy adult male volunteers. AJR Am J Roentgenol..

[36] Nazarian LN, McShane JM, Ciccotti MG, O'Kane PL, Harwood MI (2003). Dynamic US of the anterior band of the ulnar collateral ligament of the elbow in asymptomatic major league baseball pitchers. Radiology..

[37] Sasaki J, Takahara M, Ogino T, Kashiwa H, Ishigaki D, Kanauchi Y (2002). Ultrasonographic assessment of the ulnar collateral ligament and medial elbow laxity in college baseball players. J Bone Joint Surg Am..

[38] Ciccotti MC, Hammoud S, Dodson CC, Cohen SB, Nazarian LN, Ciccotti MG (2014). Stress ultrasound evaluation of medial elbow instability in a cadaveric model. Am J Sports Med..

[39.•] Ciccotti MG, Atanda A, Jr., Nazarian LN, Dodson CC, Holmes L, Cohen SB. Stress sonography of the ulnar collateral ligament of the elbow in professional baseball pitchers: a 10-year study. Am J Sports Med. 2014;42(3):544-51. 10.1177/0363546513516592. **While this is an older study, it is the largest study of mUCL evaluation in professional pitchers and should always be highlighted as a key work in this area.** 10.1177/0363546513516592 PMC4131844 24473498

[40] Bica D, Armen J, Kulas AS, Youngs K, Womack Z (2015). Reliability and precision of stress sonography of the ulnar collateral ligament. J Ultrasound Med..

[41.•] Shanley E, Smith M, Mayer BK, Bailey LB, Thigpen CA, Tokish JM, et al. Using Stress Ultrasonography to Understand the Risk of UCL Injury Among Professional Baseball Pitchers Based on Ligament Morphology and Dynamic Abnormalities. Orthop J Sports Med. 2018;6(8):2325967118788847. 10.1177/2325967118788847. **Pitchers with an absolute stress joint space of ≥5.6 mm had a 6 times greater risk of sustaining an mUCL tear requiring surgery during the subsequent season.** 10.1177/2325967118788847 PMC6088493 30116762

[42] Chalmers PN, English J, Cushman DM, Zhang C, Presson AP, Yoon S, Schulz B, Li B (2021). The ulnar collateral ligament responds to stress in professional pitchers. J Shoulder Elbow Surg..

[43] Lee GA, Katz SD, Lazarus MD (1998). Elbow valgus stress radiography in an uninjured population. Am J Sports Med..

[44] Ikuta T, Yoshioka K, Matsuzawa K, Maruyama S, Edama M (2021). Influence of continuous elbow valgus stress on the medial elbow joint space. Orthop J Sports Med..

[45.••] Yoshioka K, Matsuzawa K, Ikuta T, Maruyama S, Edama M. Changes in medial elbow joint space when elbow valgus stress is applied at different limb positions and loads in vivo. Orthop J Sports Med. 2021;9(11):23259671211045981. 10.1177/23259671211045981. **Ulnohumeral joint space was measured along with forearm muscle electromyography activity during increasing levels of valgus stress. The study found that defensive muscle contraction began above 60 N.This suggets that valgus stress above 60 N may cause defensive muscle contractions across the UHJ.** 10.1177/23259671211045981 PMC8649105 34888388

[46] Floris S, Olsen BS, Dalstra M, Søjbjerg JO, Sneppen O (1998). The medial collateral ligament of the elbow joint: anatomy and kinematics. J Shoulder Elbow Surg..

[47] Søjbjerg JO, Ovesen J, Nielsen S (1987). Experimental elbow instability after transection of the medial collateral ligament. Clin Orthop Relat Res..

[48.•] Rogers TH HA, Jacobson DS, et al. Does proximal vs distal injury location of the medial ulnar collateral ligament of the elbow differentially impact elbow stability? An ultrasound-guided and robot assisted biomechanical study. The Journal of Shoulder and Elbow Surgery. 2022 (in press). **A robot was used to impart a continuous valgus force through a full range of motion in mUCL deficient cadaveric elbows. The results showed that maximum UHJ instability occurred at 49° of elbow flexion. This suggests that 49° (or 45–50 degrees for measurement ease) is the best flexion angle to replicate the maximum stress on the mUCL during throwing.** 10.1016/j.jse.2022.03.009 35483567

[49] Hattori H, Akasaka K, Otsudo T, Hall T, Amemiya K, Mori Y, Sakaguchi K, Tachibana Y (2020). Changes in medial elbow elasticity and joint space gapping during maximal gripping: reliability and validity in evaluation of the medial elbow joint using ultrasound elastography. J Shoulder Elbow Surg..

[50] Hattori H, Akasaka K, Otsudo T, Hall T, Sakaguchi K, Tachibana Y (2021). Ulnar collateral ligament laxity after repetitive pitching: associated factors in high school baseball pitchers. Am J Sports Med..

[51] Hendawi TK, Rendos NK, Warrell CS, Hackel JG, Jordan SE, Andrews JR, Ostrander RV (2019). Medial elbow stability assessment after ultrasound-guided ulnar collateral ligament transection in a cadaveric model: ultrasound versus stress radiography. J Shoulder Elbow Surg..

[52] Gustas CN, Lee KS (2016). Multimodality imaging of the painful elbow: current imaging concepts and image-guided treatments for the injured thrower’s elbow. Radiol Clin North Am..

[53] Hultman KL, Goldman BH, Nazarian LN, Ciccotti MG (2021). Ultrasound examination techniques for elbow injuries in overhead athletes. J Am Acad Orthop Surg..

[54.•] Hattori H, Akasaka K, Otsudo T, Hall T, Amemiya K, Mori Y. The effect of repetitive baseball pitching on medial elbow joint space gapping associated with 2 elbow valgus stressors in high school baseball players. J Shoulder Elbow Surg. 2018;27(4):592-8. 10.1016/j.jse.2017.10.031. **High school baseball pitchers demonstrated significant amounts of UHJ widening from baseline after just 60 successive pitches. Prior literature has shown changes in mUCL laxity occur over the course of a pitching season. This study shows that this laxity change can occur with much short duration of load.** 10.1016/j.jse.2017.10.031 29289491

[55] Khalil LS, Jildeh TR, Abbas MJ, Klochko CL, Scher C, Van Holsbeeck M (2021). Elbow torque may be predictive of anatomic adaptations to the elbow after a season of collegiate pitching: a dynamic ultrasound study. Arthrosc Sports Med Rehabil..

[56] Tanaka K, Okamoto Y, Makihara T, Maehara K, Yoshizawa T, Minami M, Yamazaki M (2017). Clinical interpretation of asymptomatic medial collateral ligament injury observed on magnetic resonance imaging in adolescent baseball players. Jpn J Radiol..

[57] Malghem J, Omoumi P, Lecouvet FE, Vande Berg BC (2011). Presumed intraarticular gas microbubbles resulting from a vacuum phenomenon: visualization with ultrasonography as hyperechoic microfoci. Skelet Radiol..

[58] Sugimoto H, Hyodoh K, Shinozaki T (1998). Throwing injury of the elbow: assessment with gradient three-dimensional, fourier transform gradient-echo and short tau inversion recovery images. J Magn Reson Imaging..

[59] Atanda A, Averill LW, Wallace M, Niiler TA, Nazarian LN, Ciccotti MG (2016). factors related to increased ulnar collateral ligament thickness on stress sonography of the elbow in asymptomatic youth and adolescent baseball pitchers. Am J Sports Med..

[60] Atanda A, Buckley PS, Hammoud S, Cohen SB, Nazarian LN, Ciccotti MG (2015). Early anatomic changes of the ulnar collateral ligament identified by stress ultrasound of the elbow in young professional baseball pitchers. Am J Sports Med..

[61] Khalil LS, Okoroha KR, Jildeh TR, Matar RN, Fidai MS, Tramer JS, Ansok C, Scher C, van Holsbeeck M, Makhni EC, Moutzouros V (2019). Do anatomic changes found in the throwing arm after a season of pitching resolve with off-season rest? A dynamic ultrasound study. JSES Open Access..

[62] Nagamoto H, Yamamoto N, Kurokawa D, Takahashi H, Muraki T, Tanaka M, et al. Evaluation of the thickness of the medial ulnar collateral ligament in junior high and high school baseball players. J Med Ultrason (2001). 2015;42(3):395-400. 10.1007/s10396-014-0605-1. 10.1007/s10396-014-0605-1 26576792

[63] Popovic N, Ferrara MA, Daenen B, Georis P, Lemaire R (2001). Imaging overuse injury of the elbow in professional team handball players: a bilateral comparison using plain films, stress radiography, ultrasound, and magnetic resonance imaging. Int J Sports Med..

[64] Tajika T, Yamamoto A, Oya N, Ichinose T, Shimoyama D, Sasaki T, Shitara H, Kitagawa T, Saito K, Osawa T, Takagishi K (2016). The morphologic change of the ulnar collateral ligament of elbow in high school baseball pitchers, with and without symptoms, by sonography. J Shoulder Elbow Surg..

[65] Khalil LS, Meta FS, Tramer JS, Klochko CL, Scher C, Van Holsbeeck M (2021). Elbow torque is reduced in asymptomatic college pitchers with elbow laxity: a dynamic ultrasound study. Arthroscopy..

[66] Marshall NE, Keller RA, Van Holsbeeck M, Moutzouros V (2015). ulnar collateral ligament and elbow adaptations in high school baseball pitchers. Sports Health..

[67] Jacobson JA, Propeck T, Jamadar DA, Jebson PJ, Hayes CW (2003). US of the anterior bundle of the ulnar collateral ligament: findings in five cadaver elbows with MR arthrographic and anatomic comparison--initial observations. Radiology..

[68] Shukla M, Keller R, Marshall N, Ahmed H, Scher C, Moutzouros VB, van Holsbeeck M (2017). Ultrasound evaluation of the ulnar collateral ligament of the elbow: which method is most reproducible?. Skelet Radiol..

[69] Thomas SJ, Paul RW, Rosen AB, Wilkins SJ, Scheidt J, Kelly JD (2020). Return-to-play and competitive outcomes after ulnar collateral ligament reconstruction among baseball players: a systematic review. Orthop J Sports Med..

[70] Davis LC, Baumer TG, Bey MJ, Holsbeeck MV. Clinical utilization of shear wave elastography in the musculoskeletal system. Ultrasonography. 2019;38(1):2-12. 10.14366/usg.18039. 10.14366/usg.18039 PMC6323314 30343557

[71] Taljanovic MS, Gimber LH, Becker GW, Latt LD, Klauser AS, Melville DM, Gao L, Witte RS (2017). Shear-wave elastography: basic physics and musculoskeletal applications. Radiographics..

[72] Ryu J, Jeong WK. Current status of musculoskeletal application of shear wave elastography. Ultrasonography. 2017;36(3):185-97. 10.14366/usg.16053. 10.14366/usg.16053 PMC5494870 28292005

[73] Winn N, Lalam R, Cassar-Pullicino V (2016). Sonoelastography in the musculoskeletal system: current role and future directions. World J Radiol..

[74] Dirrichs T, Quack V, Gatz M, Tingart M, Kuhl CK, Schrading S (2016). Shear wave elastography (SWE) for the evaluation of patients with tendinopathies. Academic Radiology..

[75] Lin C-Y, Sadeghi S, Bader DA, Cortes DH. Ultrasound shear wave elastography of the elbow ulnar collateral ligament: reliability test and a preliminary case study in a baseball pitcher. Journal of Engineering and Science in Medical Diagnostics and Therapy. 2017;1(1). 10.1115/1.4038259.

[76] Gupta N, Labis JS, Harris J, Trakhtenbroit MA, Peterson LE, Jack RA (2019). Shear-wave elastography of the ulnar collateral ligament of the elbow in healthy volunteers: a pilot study. Skeletal Radiol..

[77] Kissenberth MJ, Thigpen CA, Bailey LB, Campbell J, Geist DJ, Schweppe ML, Wyland DJ, Hawkins RJ, Noonan TJ, Shanley E (2021). Professional pitchers display differences in UCL morphology and elbow gapping during moving valgus stress testing after UCL reconstruction. Orthop J Sports Med..

